# Clinical deterioration following arterial switch surgery due to extensive aortopulmonary collaterals, unusual condition but worth considering: case report

**DOI:** 10.1186/s43044-023-00349-2

**Published:** 2023-03-30

**Authors:** Gaser Abdelmohsen, Naif AlKhushi, Abdullah Alassiri, Osman Al-Radi

**Affiliations:** 1 Pediatric Cardiology Division, Department of Pediatrics, King Abdulaziz University, P.O Box 80215, Jeddah, 21589 Saudi Arabia; 2 Pediatric Cardiology Division, Department of Pediatrics, Cairo University, Cairo, 11562 Egypt; 3 Faculty of Medicine, King Abdulaziz University, P.O Box 80215, Jeddah, 21589 Saudi Arabia; 4 Cardiac Surgery Division, Department of Surgery, King Abdulaziz University, P.O Box 80215, Jeddah, 21589 Saudi Arabia

**Keywords:** Transposition of great arteries, Aortopulmonary collaterals, Arterial switch operation, MAPCA, D-TGA

## Abstract

**Background:**

The occurrence of major aortopulmonary collateral arteries (MAPCAs) is infrequent in patients with D-transposition of great arteries (D-TGA) with intact ventricular septum (IVS). Hemodynamically significant MAPCAs may complicate the postoperative course of these patients after arterial switch operation (ASO).

**Case presentation:**

We present a rare case of neonatal D-TGA-IVS associated with *extensive* MAPCAs. After the ASO, the patient developed pulmonary hemorrhage, chest wall edema, and deterioration of lung compliance with the need for high-frequency ventilation (HFV). The patient also had a significant capillary leak with skin edema, high chest tube drainage, and high peritoneal drainage. Cardiac catheterization revealed *extensive* MAPCAs supplying the whole lung segments. After the catheter closure of most of these MAPCAs, the patient had clinical improvement.

**Conclusions:**

Although the occurrence of MAPCAs with D-TGA-IVS is infrequent, clinicians should suspect their presence in cases with unexplained heart failure, pulmonary hemorrhage, or cardiovascular compromise after ASO. Catheter closure of MAPCAs is feasible with an acceptable short-term outcome.

**Supplementary Information:**

The online version contains supplementary material available at 10.1186/s43044-023-00349-2.

## Background

Arterial switch operation (ASO) is the standard surgical treatment for patients with D-TGA in the early neonatal period. Although the presence of MAPCAs in cases with D-TGA-IVS is infrequent, and most of these MAPCAS have no clinical significance, some patients may have serious complications related to significant MAPCAs. MAPCAs can cause significant left-to-right shunting causing pulmonary edema, pulmonary hemorrhage, heart failure symptoms, and tissue hypoperfusion (gut ischemia and oliguria) secondary to significant steal from the systemic flow [1–4]. We present a rare case with D-TGA-IVS with extensive MAPCAs presented with unexplained stormy postoperative course after ASO that improved after catheter closure of these MAPCAs.

## Case presentation

A full-term, 14 days old and 2.8 kg female baby was referred to our cardiac center as a case of D-TGA-IVS. Immediately after birth, the patient had cyanosis, and oxygen saturation was 75; prostaglandin E1 was started. A detailed echocardiogram revealed D-TGA with IVS, a large patent ductus arteriosus (PDA) of 4 mm, with a 5-mm atrial septal defect (ASD) and normal coronary anatomy (from posterior facing aortic sinuses).

**Fig. 1:**
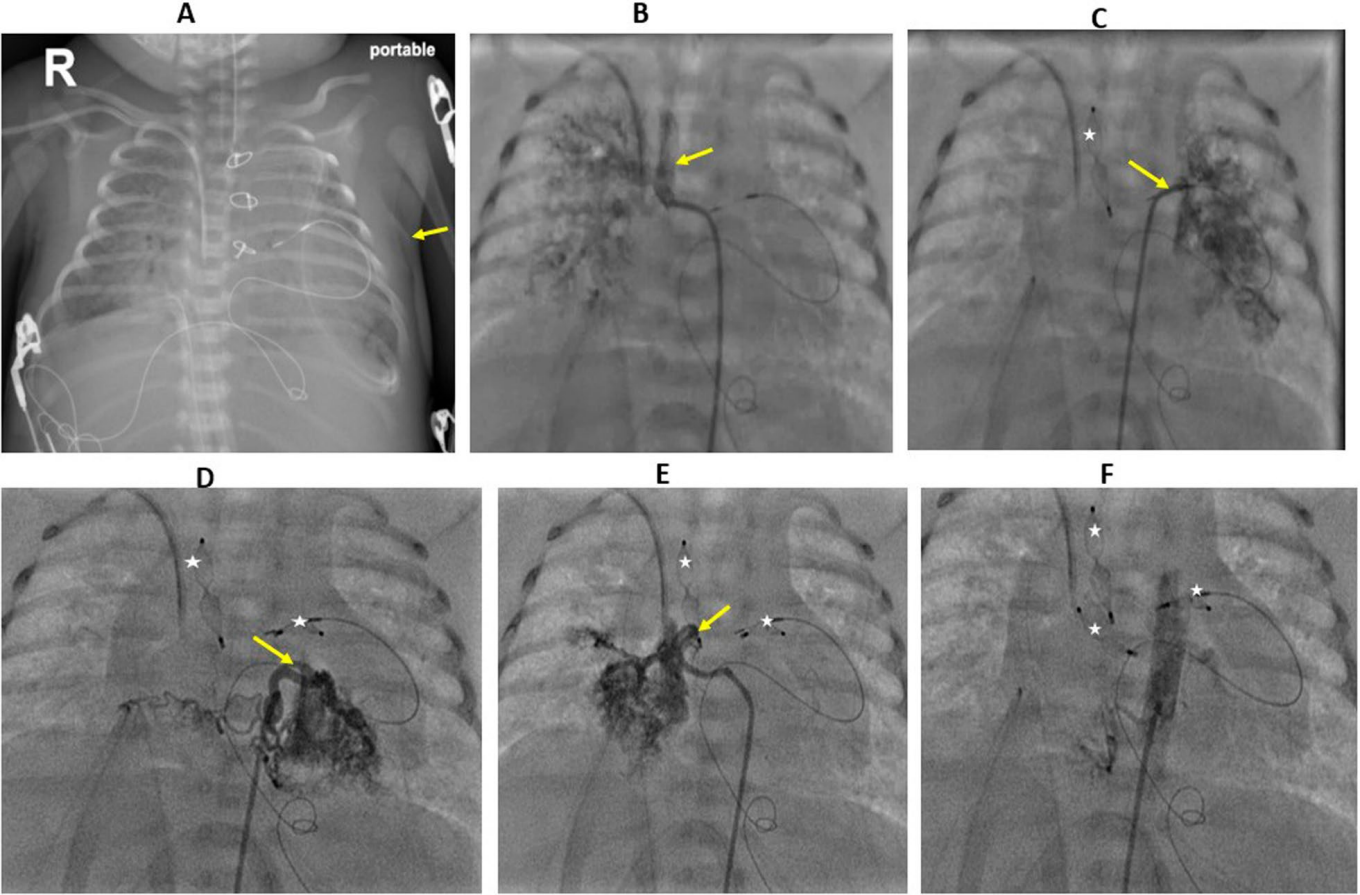
Roentgenogram and angiography before and after the closure of MAPCAs: **A** chest X-ray after ASO showed pulmonary edema with significant skin edema (yellow arrow). **B** selective angiography showing a large MAPCA supplying the right upper lung lobe (yellow arrow). **C** selective angiography showing a large MAPCA supplying the left upper lung lobe (yellow arrow) with vascular plug seen closing the MAPCAs of right upper lobe (white asterisk). **D** selective angiography showing MAPCA supplying the left lower lung zone with vascular plugs seen occluding MAPCAS for upper left and right lungs (white asterisks). **E** selective angiography showing large MAPCA supplying the right lower lung lobe (yellow arrow). **F** Successful closure of 3 MAPCAs using three vascular plugs (white asterisks). ASO: arterial switch operation, MAPCAs: Major aortopulmonary collateral arteries

**Fig. 2:**
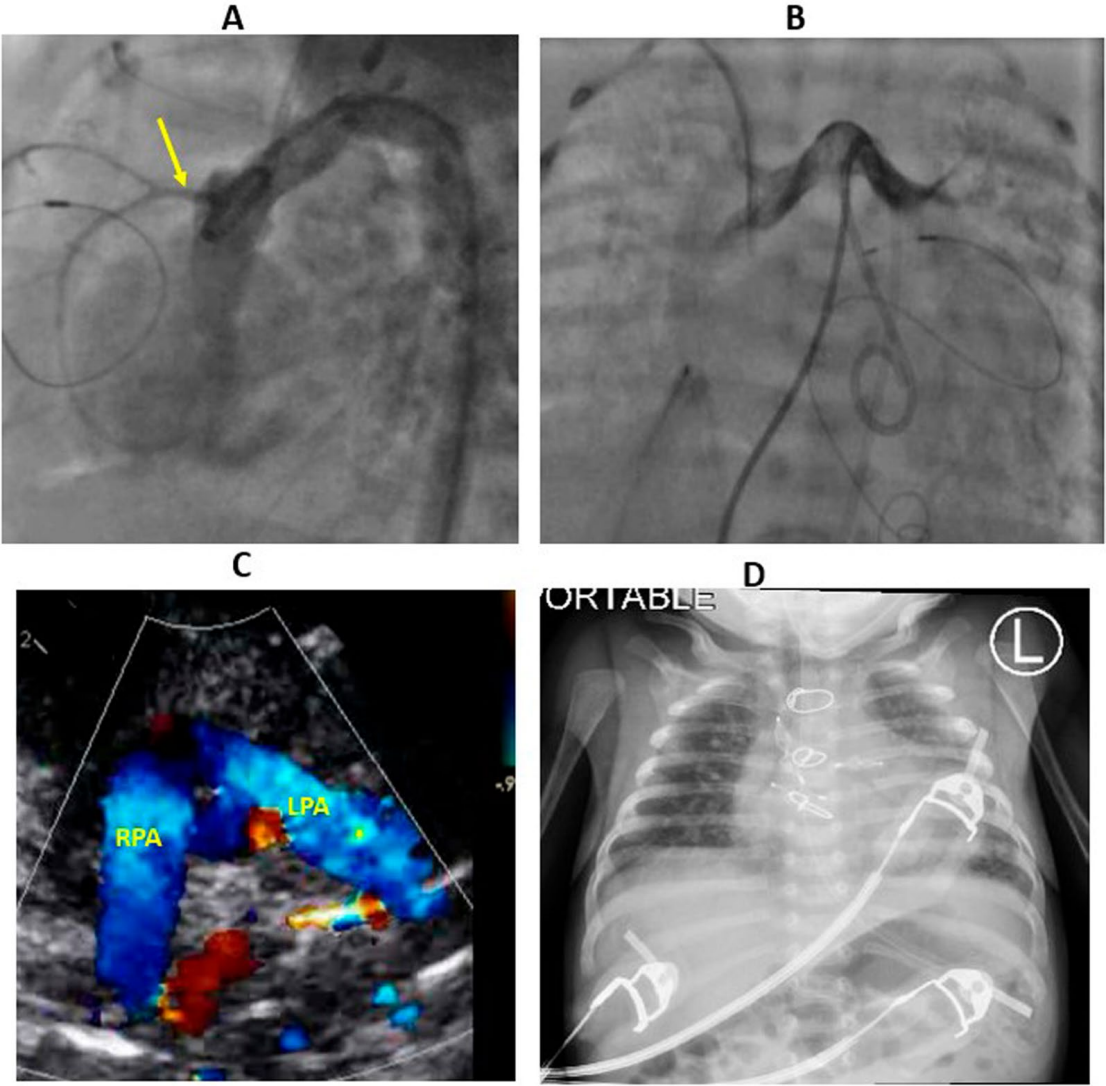
Roentgenogram, angiography, and echocardiography after ASO. **A** aortic angiography showing normal right coronary artery (yellow arrow). **B** Pulmonary angiography showing patent pulmonary arteries. **C** Parasternal short axis echocardiographic view showing the Lecompte with patent pulmonary arteries.** D** chest X-ray is done after successful closure of MAPCAs, showing improvement of pulmonary edema and skin edema. ASO: arterial switch operation, MAPCAs: Major aortopulmonary collateral arteries. *RPA* right pulmonary artery, *LPA* left pulmonary artery

Uneventful arterial switch operation was done successfully. At surgery, no evidence of unusual findings was encountered. The bypass time was 71 min, and the cross-clamp time was 54 min. The patient came from the operating room with a closed chest and on Epinephrine 0.05 mcg/kg/minute and Milrinone 0.5 mcg/kg/minute. Following arrival from the operating room, the patient had developed significant pulmonary hemorrhages and required a high ventilator setting. The patient received blood products and shifted to HFV. Chest X-ray showed significant lung plethora (Fig. 1A), and echocardiography revealed a fair cardiac function (ejection fraction 55%) with no regional wall abnormalities and no stenosis of the main pulmonary artery, pulmonary branches, or aorta. The second day after surgery, the patient started to develop chest wall edema, hypotension, oliguria, and CO2 retention, so the chest was opened, Epinephrine was increased to 0.07mcg/kg/minute, Milrinone to 1 mcg/kg/minute, and Norepinephrine to 0.05mcg/kg/minute and Furosemide infusion was added. After opening the chest, hemodynamics showed some improvement. The patient was then shifted to the catheterization laboratory on the 7th day after surgery for hemodynamic evaluation, coronary angiography, and looking for any MAPCAs. Aortic angiography revealed extensive 4 MAPCAs supplying both lungs (Fig. 1B–E). A 4 French Judkins right catheter (JR4) was advanced through the femoral artery and thoracic aorta; the catheter was then advanced to the collaterals, and an Amplatzer type IV vascular plug was backloaded into the catheter, advanced deeply into the collateral, and then released. Three MAPCAs were successfully closed with three Amplatzer vascular plugs type IV (6 mm, 4 mm, 4 mm), but the fourth one (supplying the left lower lung lobe) could not be closed due to its highly tortuous anatomy (Fig. 1B–F, Additional file 1: Video 1). Pulmonary angiography and aortic angiography revealed no abnormalities (Fig. 2A, B).

After the closure of MAPCAs, the chest wall edema persisted, causing a significant decrease in lung compliance with prolonged ventilation. After getting an excellent negative balance through diuresis, chest wall edema and lung compliance improved gradually. Then, the patient was progressively weaned from mechanical ventilation till extubation (Fig. 2D); the chest was closed 15 days after the surgery, and the patient was extubated 12 days after chest closure. Two weeks after extubation patient was discharged home in good condition. The patient came for a follow-up in the outpatient clinic two weeks after discharge. The patient was doing fine, with no history of feeding intolerance, dyspnea with feeding, or interrupted feeding, oxygen saturation was 98%, and echocardiography revealed good cardiac function, no regional wall abnormalities, no significant supravalvular aortic stenosis or branch pulmonary stenosis, trivial neo-aortic insufficiency.

## Discussion

MAPCAs represent remnants of the embryonic splanchnic circulation; usually, they are clinically insignificant and involute with time. They are believed to persist in congenital cardiac lesions associated with decreased pulmonary blood flow and lower oxygen saturations, as in patients with pulmonary atresia and ventricular septal defect [5, 6]. MAPCAs are rare in patients with D-TGA; in some reports, 2% of patients with D-TGA have MAPCAs, especially those with lower oxygen saturations [1]. Significant MAPACs often cause significant diastolic flow reversal in the descending aorta detected during echocardiography. However, echocardiography is limited in detecting MAPCAs, especially in the preoperative period. The presence of the PDA can also cause significant diastolic aortic flow reversal, as in our patient. Computed tomography and angiography are essential for evaluating MAPCAs, but they are rarely done preoperatively unless abnormal coronary anatomy is suspected. During cardiac surgery, MAPCAs may be expected when left atrial venous return is in excess [1, 3].

All clinical and echocardiographic signs indicating the presence of MAPCAs in our patient were insufficient. The preoperative patient's oxygen saturation was not very high (75%), there was no significant preoperative heart failure or low cardiac output status due to substantial steal, and the large ductus could explain the retrograde flow in the proximal descending aorta seen during echocardiography. Finally, the left atrium was not accessed during cardiac surgery to determine if there was an excessive pulmonary venous return. Following arterial switch operation, adding Milrinone and a high fraction of inspired oxygen (FIO2) may significantly decrease pulmonary vascular resistance, resulting in more left-to-right shunting and a stormy postoperative course.

Although ASO has an excellent outcome, significant MAPCAs can complicate the postoperative course, as in our patient. Hemodynamically significant MAPCAs are commonly present after ASO due to the physiological decrease in pulmonary vascular resistance (PVR). PVR gradually decreases after birth; with each reduction in PVR, pulmonary blood flow increases, resulting in pulmonary plethora, heart failure, pulmonary hemorrhage, stiff lungs, and left chamber dilatation.

In this case report, during the first few days after arterial switch surgery, we suspected that the patient's conditions were related to capillary leak syndrome, which rarely occurs in some neonates after cardiopulmonary bypass. However, delayed improvement prompted us to perform cardiac catheterization for hemodynamic evaluation and angiography. The high inotropic support might maintain the patient's hemodynamic stability till the closure of MAPCAs. Notably, Norepinephrine was added in response to the marked capillary leak and third space loss.

MAPCAs with hemodynamic significance usually require catheter closure. Catheter closure of significant MAPCAs after ASO is associated with good short-term outcomes. Previous studies found that patients with ASO and MAPCAs that did not require cardiac catheterization had shorter mechanical ventilation duration, intensive care unit stay, less inotropic support, and shorter lengths of stay [1, 2, 7, 8]. This could explain our patient's chaotic postoperative course.

## Conclusions

While aortopulmonary collateral arteries (MAPCAs) are infrequently associated with D-TGA-IVS, their presence may worsen the postoperative course after arterial switch surgery. Significant MAPCAs should be suspected after the arterial switch if the postoperative period is complicated by acute cardiovascular decompensation, respiratory failure, or postoperative pulmonary hemorrhage. Significant MAPCAs can be closed through cardiac catheterization with a favorable outcome.

## Supplementary Information

**Additional file 1.** **Video 1**. shows the catheter closure of the MAPCAs. MAPCAs: Major aortopulmonary collateral arteries.

## Data Availability

All data and materials will be uploaded as per the needs of the editor/reviewer or the readers as per their request.

## Abbreviations

ASD: Atrial septal defect
ASO: Arterial switch operation
FIO2: Fraction of inspired oxygen
HFV: High-frequency ventilation
IVS: Intact ventricular septum
LPA: Left pulmonary artery
MAPCAs: Major aortopulmonary collateral arteries
PDA: Patent ductus arteriosus
PVR: Pulmonary vascular resistance
RPA: Right pulmonary artery
TGA: Transposition of great arteries
